# Measurement and Evaluation of Local Surface Temperature Induced by Irradiation of Nanoscaled or Microscaled Electron Beams

**DOI:** 10.1186/s11671-018-2821-x

**Published:** 2019-01-22

**Authors:** Zhenhai Wang, Lijiang Gui, Danhong Han, Zhuang Xu, Li Han, Shengyong Xu

**Affiliations:** 1 Key Laboratory for the Physics & Chemistry of Nanodevices, and Department of Electronics, Peking University, Beijing, 100871 China; 2 Department of Micro-Nano Fabrication Technology, Institute of Electrical Engineering, Chinese Academy of Sciences, Beijing, 100190 China

**Keywords:** Electron beam, Local temperature, Thin film thermocouple, Nanoscale thermometry, Transmission electron microscopy, Scanning electron microscopy, Vaporization, Melting point, Energy converting

## Abstract

Electron beams (e-beams) have been applied as detecting probes and clean energy sources in many applications. In this work, we investigated several approaches for measurement and estimation of the range and distribution of local temperatures on a subject surface under irradiation of nano-microscale e-beams. We showed that a high-intensity e-beam with current density of 10^5-6^ A/cm^2^ could result in vaporization of solid Si and Au materials in seconds, with a local surface temperature higher than 3000 K. With a lower beam intensity to 10^3-4^ A/cm^2^, e-beams could introduce local surface temperature in the range of 1000–2000 K shortly, causing local melting in metallic nanowires and Cr, Pt, and Pd thin films, and phase transition in metallic Mg-B films. We demonstrated that thin film thermocouples on a freestanding Si_3_N_4_ window were capable of detecting peaked local surface temperatures up to 2000 K and stable, and temperatures in a lower range with a high precision. We discussed the distribution of surface temperatures under e-beams, thermal dissipation of thick substrate, and a small converting ratio from the high kinetic energy of e-beam to the surface heat. The results may offer some clues for novel applications of e-beams.

## Introduction

E-beams have been applied as probes and clean energy sources in a variety of practical applications, such as imaging surface morphology, analyzing crystalline structures, producing lithography patterns, depositing thin films, etc. In these practical applications, local surface heating effect under irradiation of e-beams is an interesting issue. For some applications, in e-beam lithography for instance, this e-beam-induced surface heat may cause distortion of designed patterns at the nanoscale in the e-beam writing process, thus resulting in failure of the final devices [1–3]. In some other applications, the local surface heating effect is utilized for thin film deposition [4], annealing [5–7], or even sculpting of nanomaterials [8, 9]. Yet to date, precise measurement and evaluation of the local surface temperatures under e-beam irradiation remain as a technical challenge [10–12].

When the “free electrons” in metals are given high kinetic energy, e.g., by heating or a high electric field, they can run out of the metal bulk into free space. Electron beam has been used widely in welding [13–16], melting [17], edge cutting-off [18], surface treatment [19, 20], and physical vapor deposition [21]. Nowadays, much recent progress in metal additive manufacturing processes using e-beam melting has been made [22–26]. It has been attracting increasing attention on making use of e-beam for selectively melting the metal powder. The e-beams discussed in this work refer to those emitting from electron guns made from tungsten wires, LaB_6_ crystal tips, or W crystal tips [27, 28]. After accelerating under a high electric field, an e-beam becomes a pure energy source with an average of 5–30 keV per electron in scanning electron microscopes (SEMs), or of 100–300 keV per electron in normal transmission electron microscopes (TEMs).

The phenomena associated with the reentry process of a free e-beam in vacuum when it is guided to enter a clean solid surface in a SEM or a TEM are well studied. When a high-energy (5–300 keV) e-beam reaches a solid surface, it usually generates many secondary electrons, back-scattering electrons, Auger electrons, etc. It may also induce excitation of atoms under irradiation, and cause “knock-on effect” that removes some surface atoms, and may break local crystalline structure thus induce disorders and defects [29, 30]. Besides, these electron-solid interactions cause increment of local temperature of the subject under the e-beam irradiation.

In general, the increment of local temperature results from converting kinetic energy of the e-beam to the subject under irradiation. Previous studies have shown that the local temperatures under a high-intensity e-beam (HIEB) could be very high. A HIEB in a TEM could drill nano-holes, cut nano-bridges in nanowires (NWs), and to weld two NWs forming a junction [8]. With careful operation procedures, one could use an e-beam to make a bridge of single-atom chain of carbon from a continuous carbon thin film [9]. Using e-beams as in situ energy sources, one could obtain superconducting MgB_2_ phase from a multilayered [Mg-B]_n_ “superlattice” film [5–7].

However, it remains a controversial issue: What is the exact local temperature induced by an e-beam? Technically, it is hard to directly measure the local temperature induced by a focused e-beam in a TEM or a SEM. Several attempts were made to solve this problem [31, 32]. For instance, the temperature profile at the surface of a resist film under an e-beam irradiation was measured with submicron thin film thermocouples (TFTCs). Local temperature profiles with a spatial resolution of nanometer were obtained with a scanning transmission electron microscope (STEM) and electron energy loss spectroscopy (EELS) [33]. Direct measurement of local temperature in nanoscale environment was performed in TEM with a combined electron energy gain and loss spectroscopy [34]. Using the parallel beam electron diffraction method, the local temperature in a TEM was indirectly measured from the change in scattering angle induced by thermal expansion [35]. Taking advantage of a TEM and solid-liquid phase transition of metallic islands, nanoscale thermal images were obtained, whose resolution surpass the limits of a thermal microscopy based on infrared imaging technique [36]. Using this method, the mechanism of remote Joule heating of a silicon nitride substrate by a single multi-walled carbon nanotube had been discovered [37]. Moreover, the micro-thermometers, based on the metal-insulator transition, could give quantitative evaluation of electron beam heating in detail [38].

Yet these attempts were only applicable to a low-temperature range. In this work, by analyzing the structure change of the material before and after the irradiation, and by directly measuring the local temperature with devices and measurement techniques we developed [39, 40], we analyzed the heating effects induced with nanoscale e-beams for a temperature range of six orders of magnitudes with in situ experiments in TEM and SEM. For temperatures higher than 10^3^ K, we estimated the local temperatures under an e-beam with morphology changes in semiconductor and metallic nanowires, as well as nano-stripes of metallic thin films. For temperatures lower than 10^3^ K down to a friction of 1 K, we measured the local temperatures under an e-beam with micro-/nano-TFTC devices fabricated on freestanding Si_3_N_4_ films. The whole spectrum for local maximum temperature versus incident intensity of e-beam may offer a valuable reference for novel applications involving e-beam processes.

## Experimental Details

Si nanowires (NWs) used in this work were fabricated with a chemical vapor deposition (CVD) process as described earlier [8]. Cu NWs, Au NWs, and Ag NWs were fabricated with electrochemical deposition process on porous anodized alumina oxide substrate as described earlier [41].

Pt-Cr thin film thermocouples were fabricated with standard cleanroom procedures and thin film deposition techniques as reported previously [40]. In this work, Cr thin film was deposited with a magnetron sputtering system (PVD75, Kurt J. Lesker, USA) in Ar gas. The patterns of Cr appeared after lift-off process and the patterns of Pt aligned to Cr markers were manufactured with the same parameter. A 3-nm-thick Cr was deposited in advance as adhesion layer for Pt layer. For the Pt-Cr TFTC arrays, Pt and Cr thin films with thicknesses of 90 nm and 50 nm, respectively, were measured with a step profiler. On each 4-in wafer, we have designed identical 16 dies, arranged in a 4 × 4 array. Each die had a size of 16 mm × 16 mm and it consisted of one TFTC array device. Each TFTC array consisted of 24 TFTCs, which had junction size ranged from 2.0 μm × 2.5 μm to 8.0 μm × 8.5 μm. The resistances of TFTCs, ranging from 0.7 to 75.6 kΩ, were obtained by digital multimeter (2400, Keithley) for TFTC with different sizes. The thermopower of the TFTCs were calibrated to be 15.0 ± 0.3 μV/K on a homemade platform.

For the thin film micro-device fabricated on a freestanding Si_3_N_4_ window which was on a Si (100) substrate, 400-nm-thick silicon nitride layers were deposited on both sides of Si (100) wafer by low-pressure chemical vapor deposition (LPCVD) technique and showed excellent mechanical properties. After TFTC devices on the front surface were fabricated, square etching-windows were patterned and etched on the backside of the wafer, a wet-etching process was performed to etch through the Si wafer from the backside, leaving a freestanding Si_3_N_4_ thin film window with pre-patterned TFTC arrays for thermal measurements in a SEM.

The focused ion beam (FIB) milling experiments were conducted on the FIB/SEM dual beam 820 system, which reduced the TFTC junction size from 5.0 × 5.0 μm^2^ to 1.0 × 1.0 μm^2^. An Ga^+^ ion beam, whose beam current was 12 pA with an accelerating voltage of 30 KV, was used in the process of reduction.

In our in situ annealing experiments of Mg/B thin films, we took advantage of commercial magnesium slugs (99.99%) and boron (99.5%) to be the evaporation sources for deposition of Mg–B multilayer films. The base vacuum in the deposition chamber was about 5.0 × 10^− 6^ Pa. A 15-nm-thick magnesium layer was served as the first layer, which was deposited by electron beam evaporation at room temperature on 6H–SiC (0001) substrates in a Balzers UTT 400 ultra-high vacuum (UHV) coater. After that, a 10 nm B layer was deposited on the first layer. Mg and B layers, deposited alternately on substrates, were the precursor films with a multilayered structure of [B(10 nm)/Mg(15 nm)]_*N* = 4_ on SiC substrate. The total thickness of the Mg–B multilayer was 100 nm, monitored by a quartz oscillator in situ. The thickness ratio of Mg:B = 3:2 (15 nm:10 nm = 3:2) can satisfy the composition of Mg:B = 1:2. The top 10-nm-thick boron layer was served as a film cap to reduce loss of Mg to a certain extent during annealing. The precursor films were post-annealed in an EBW-6 electron beam welder with a vacuum pressure of 5.0 × 10^− 3^ Pa. The accelerating voltage of an annealing electron beam was 40 KV with a beam current of 3 mA. The diameter of an electron beam was 1.40 cm and the annealing duration was 0.1–1.0 s.

The HIEB experiments in TEM were carried out on a 200-kV Jeol 2010F field-emission TEM. The e-beam current was measured to be ~ 5 nA with a Faraday cup. At the e-beam diameter of 0.5–1.0 nm, a nominal current density of (0.6–2.5) × 10^6^ A/cm^2^ was yielded on the specimen. TEM specimens were prepared on lacey carbon grids from suspensions of the NWs in ethanol. The procedures employed for the patterning and welding of NWs followed the description in ref. [9]. SEM experiments were performed in an EBW-3H vacuum electron-beam welder and a field emission SEM (FEI QUANTA 600F). Complicated wiring and connection between the device in the SEM vacuum chamber and measurement instruments outside vacuum chamber were specially designed and realized. Weak voltage signal outputs of the TFTC under irradiation of the e-beam of SEM at different spot size (from 1 to 7, a.u.) and accelerating voltage (from 2 to 30 keV) were measured with a homemade multiplexer and a Keithley 2182A nanovoltmeter [39].

## Results

**Fig. 1:**
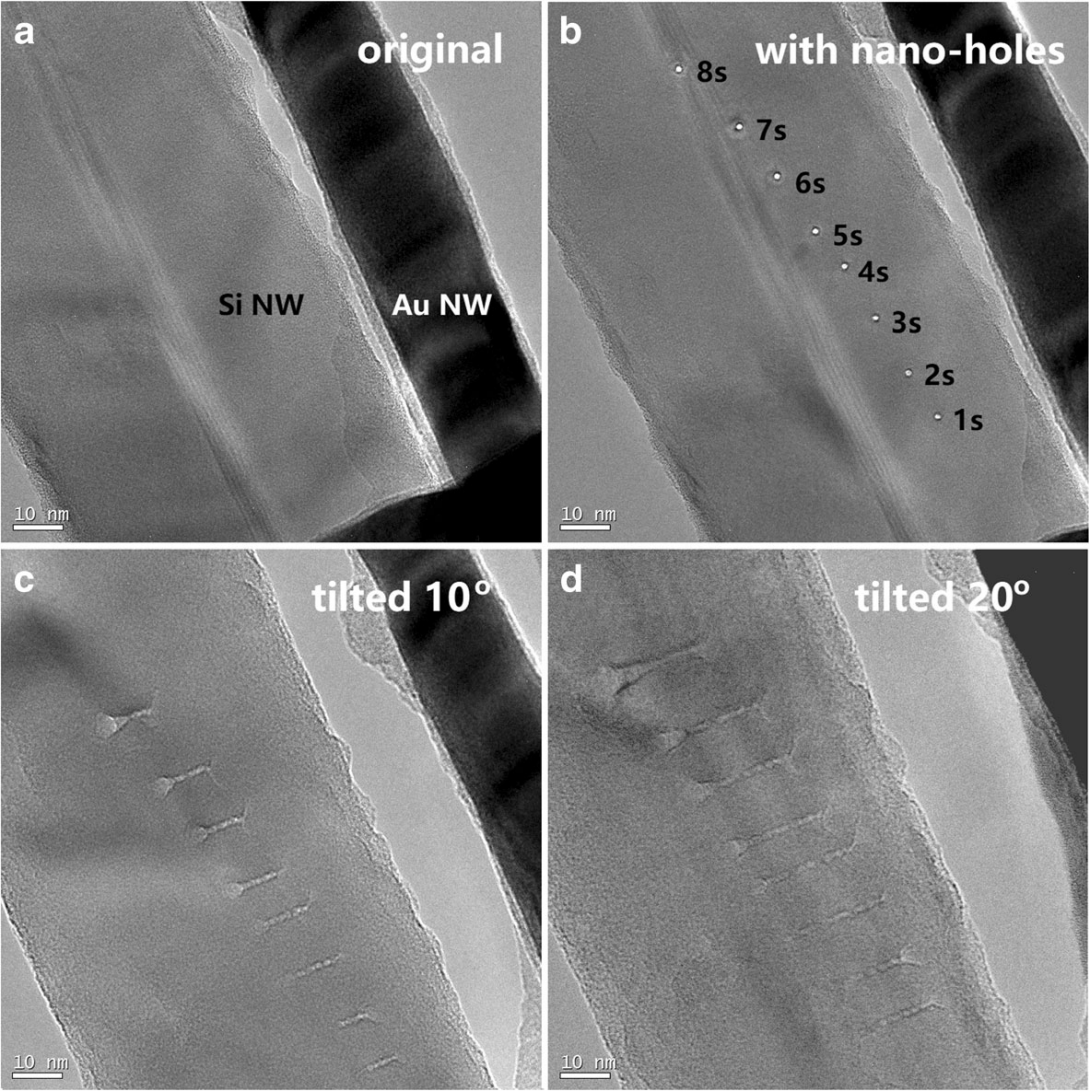
TEM morphology images showing eight 1-nm holes drilled in a 60-nm-diameter single crystalline Si NW. This is done in 1–8 s, respectively, with a 0.5–1.0-nm-diameter HIEB of current 5 nA in a 200 kV TEM. **a** The original Si NW together with a 25-nm-diameter Au NW (appearing black in the image). **b** An image after eight nano-holes have been created by a HIEB. **c**, **d** Images of the same sample after in situ tilting for 10.0° and 20.0°, respectively

We first invested the up-limit of the local temperature, ***T***_max_, that a nanoscale e-beam in a TEM could induce on a subject surface. Dozens of experimental evidences showed that atoms in surface layers of a solid NWs could be instantly vaporized under irradiation of a HIEB [8, 42], indicating that the corresponding *T*_max_ values were higher than the vaporization temperatures of the subject materials. Figure 1 presents a typical result taken in a TEM (Jeol 2010F) at operation voltage of 200 kV and e-beam current of 5 nA. The e-beam was focused into a diameter as small as 0.5–1.0 nm, and its intensity was high enough to drill 1-nm-diameter holes in a 60-nm-diameter Si NW in less than a second. Figure 1a is the original Si NW, where a 25 nm Au NW is located in parallel as a reference in tilting operation. Figure 1b shows the eight nano-holes on the Si NW made with the nanoscale e-beam in 1, 2, 3, 4, 5, 6, 7, and 8 s, respectively. The holes all have similar diameter: ~ 1.0 nm. Figure 1c, d shows side-view of the holes for the same Si NW after the sample has been tilted in situ for 10.0° and 20.0°, respectively. One sees that after irradiating for 1 s with the 0.5–1.0 nm diameter e-beam, a hole has been drilled through the whole Si NW. With increasing irradiating duration from 1 to 8 s, the diameter of front opening and bottom opening both enlarged, while the central region of the through holes remains a similar 1 nm diameter, thus appearing as not changing in the top view shown in Fig. 1b.

The fact presented in Fig. 1 was that Si atoms in the focal point local region under the 1-nm-diameter HIEB were totally vaporized. Similarly, such a 1-nm-diameter HIEB was capable of drilling holes in an Au NW [8]. A logical conclusion is that the local temperature should be higher than the boiling points of the materials, here, Si or Au. As shown in Table 1, the boiling points are 3173 K for Si and 3081 K for Au. In both cases, the local temperatures under a HIEB were higher than 3000 K. Previous studies have shown that the melting point of a nanomaterial is slightly lower than that of the bulk [43–45], yet at the size of 20–100 nm, this reduction in melting point is expected not remarkable.

**Table 1:** Vaporization and melting points of materials used in this work

Material	Melting point (K)	Boiling point (K)	Thermal conductivity (W/m K) at 300 K
Si	1687	3173	148
Au	1337	3081	317
Ag	1234	2485	429
Cu	1358	2835	401
Cr	2130	2945	90.7
Pt	2045	4098	71.6
Pd	1825	3237	71.8

Here, as the local temperature was so high that no method is applicable to directly measure the real temperature. For contact thermal sensors, the local temperature was higher than the melting or even the boiling point of the sensors. For noncontact luminescence methods, not only the size of the local area was too small for an optical fiber but also the whole process occurred too fast for reliable optical measurements.

The estimated high local temperature over 3000 K may cause argument. One may argue that instead of vaporization caused by thermal heating, the removal of local Si atoms in the eight nano-holes shown in Fig. 1 was caused by the “knock-on effect.” If drilling nano-holes with a HIEM was truly a knock-on effect, then reducing the flux of HIEM by enlarging its beam diameter, one may continuously observe removal of surface atoms in a long time scale. But what was observed was the existing threshold for the beam intensity, lower than which, drilling process in Si NWs, Au NWs, etc. could not be carried out [8]. Considering that the kinetic energy of each electron in a 200 kV TEM is around 10^−14^ J, which is larger than the binding energy per Si atom by three orders of magnitude, the vaporization and knocked-on effect have synergic effects in a “drilling” process. Therefore, by defining the *nominal local temperature* as a parameter that is proportional to the average kinetic energy of local particles (here, Si atoms), the *nominal local temperatures* at the eight hole regions were truly higher than the boiling point of Si.

Previously, it was reported that when the beam current density was in the range of 10^3–5^ A/cm^2^, an e-beam in a TEM could be applied to introduce local melting in freestanding NWs, e.g., Au NWs, Cu NWs, etc., in minutes [8]. The observed melting effects unambiguously indicated that the local temperatures on these metal surfaces were in the range of 1000–2000 K, as listed in Table 1.

**Fig. 2:**
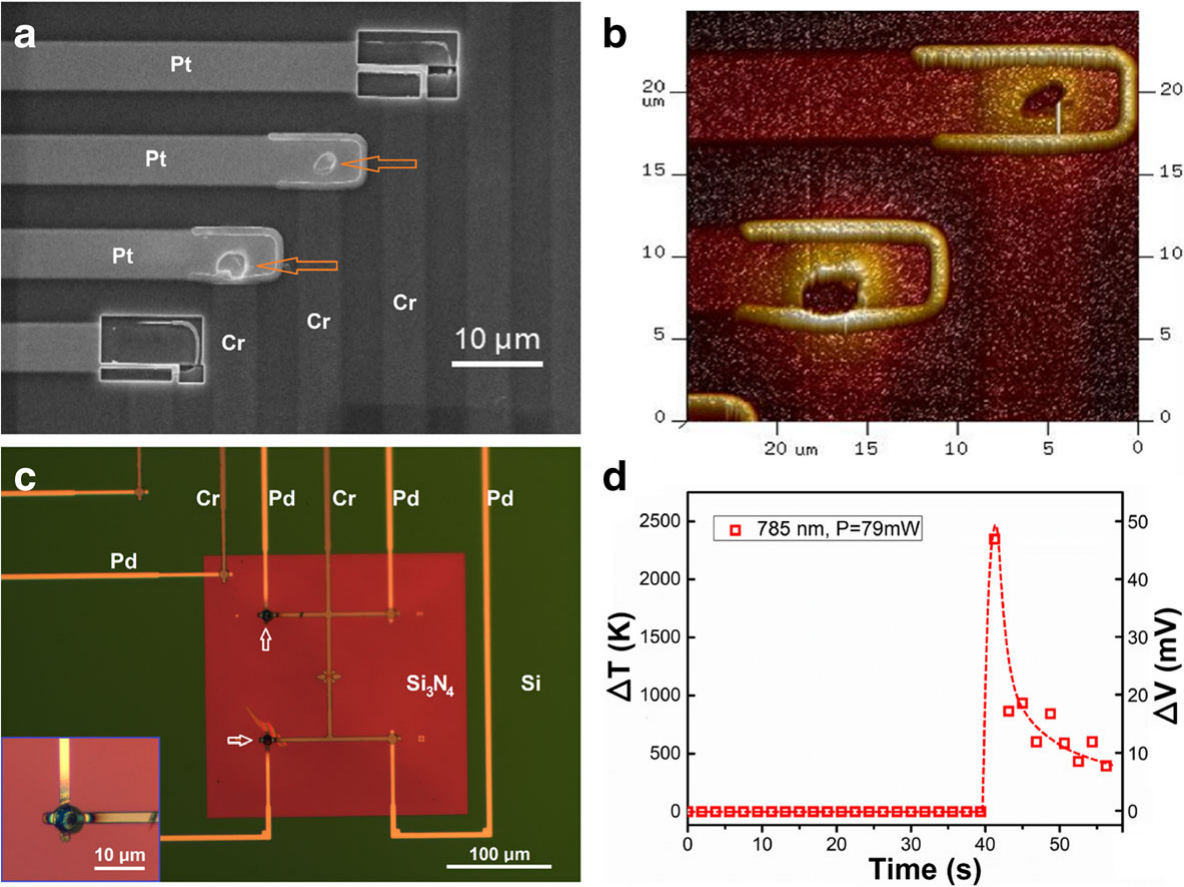
Local melting effects observed in a SEM. **a** SEM image of a Pt-Cr TFTC array sample, showing two holes (highlighted with red arrows) were made by e-beam irradiation at the junction regions of two Pt-Cr TFTC sensors. **b** AFM image of the same two junctions, showing detailed 3D information of the two holes. **c** Four Pd-Cr TFTC sensors made on a 400-nm-thick, freestanding Si_3_N_4_ thin film window. Two TFTCs (highlighted with white arrows) at the left wide of the window were burnt with a focused 785 nm laser. **d** The corresponding output peak of the Pd-Cr TFTC when it was burnt with the laser

In this work, we observed that when the beam intensity was high enough and irradiation time last for minutes, e-beams in a SEM could also induce local melting effect for Pt and Cr thin films deposition on Si, as typically presented in Fig. 2. From the SEM image (Fig. 2a) and atomic force microscope (AFM) image (Fig. 2b) of the same sample after irradiation of a high-intensity e-beam in a SEM, one sees holes and protrusions at the junction regions of two Pt-Cr TFTCs (highlighted with red arrows). The melting temperatures of Cr and Pt were 2130 K and 2045 K, respectively. Our experimental results implied that the local temperatures of surface under the irradiation of e-beam were higher than the melting temperatures of these metals (~ 2000 K) [46]. The protrusions that occurred at the junction edges a few microns away from the melting holes were probably formed by diffusion and accumulation of the Pt and Cr atoms. However, we failed to obtain the real-time local temperature values with the same Pt-Cr TFTC sensors under irradiation. Instead of showing a local temperature increment of 2000 K, we measured an increment of less than 100 K. As discussed later, this huge difference was caused by thermal dissipation of the thick Si substrate and the big size of the TFTC sensor.

With the concern of device reliability for high temperature measurement, we provided our referred experiments with high-energy lasers, whose heat focusing on the junction is enough to offset the thermal dissipation. Figure 2c shows our Pd-Cr TFTC devices made on a 400-nm-thick freestanding Si_3_N_4_ thin film window. The Pd-Cr TFTC made with the same process parameter of Pt-Cr TFTC was used to measure the Medical laser by our teammates, instead of Pt-Cr TFTC, and significant results were referred here to confirm the high temperature reliability of this type device [47]. With irradiation at the focal point of a 79 mW (power), 785 nm (wavelength) laser for 2 s, two Pd-Cr junctions were burnt (highlighted with white arrows). At the meantime, the device showed an output peak near 50 mV. By using our calibration results obtained at room temperature which was around 20 μV/K, this represents a nominal peak temperature of ~ 2400 K, as shown in Fig. 2d. But 2400 K is higher than the melting point of the Pd film, 1825 K. We attributed this error to a changing Seebeck coefficient of Pd and Cr at high temperatures. Nevertheless, our results indicated that our TFTC sensors made on Si_3_N_4_ thin film window were applicable for measuring local temperatures up to a value close to the melting points of the metallic stripes, i.e., 1800 K.

**Fig. 3:**
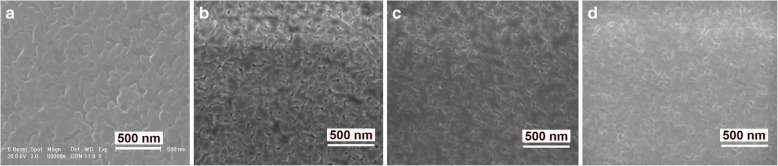
SEM micrographs of [B(10 nm)/Mg(15 nm)]_*N* = 4_ multilayers on SiC substrate annealed with HIEB in a SEM with the annealing currents of **a** 0 mA, **b** 9.9 mA, **c** 10.7 mA, and **d** 12.8 mA, respectively

The up-limit of local temperature ***T***_max_ in a SEM was also revealed with our experiments on annealing of [Mg-B]_N_ multilayered thin films. The results showed that high-intensity e-beams could induce phase transition in amorphous [Mg-B]_N_ multilayered thin films within 1 s. As a result, an amorphous multilayer was partially turned into a MgB_2_ superconducting phase [5–7]. Figure 3 and Table 2 present some typical results. The precursor films, denoted by [B(10 nm)/Mg(15 nm)]_*N* = 4_, were prepared with a total thickness of 100 nm. The accelerating voltage of the annealing e-beam was 40 kV, with the beam currents of 9.9 mA, 10.7 mA, and 12.8 mA, respectively. The SEM images of the samples revealed that different annealing currents could result in different roughness on the film surface, as shown in Fig. 3. At a sample area of 10 × 10 μm^2^, the root mean square (RMS) roughness was measured to be 3.11 nm, 3.56 nm, and 7.53 nm, respectively, for these samples. The superconducting transition temperatures ***T***_c_ of these samples were found to be 35.1 K, 35.8 K, and 36.3 K, respectively (Table 2). It implied that annealing temperature was crucial for the evaporation of Mg, the diffusion into B layers, and the reaction rate with B. A larger current could bring about a higher annealing temperature, which could lead to a more sufficient reaction. According to the phase diagram of MgB_2_, the minimum required temperature for a phase transition for forming superconducting MgB_2_ was 900–1000 K. Therefore, the e-beams had induced local temperatures around 900–1000 K, or even higher. This result was consistent with the results shown in Fig. 2.

**Table 2:** Annealing parameters and measured properties of Mg-B thin films

Sample No	Annealing current I (mA)	Energy density E (J/cm^2^)	Critical point *T*_c_ (K)	Mean roughness (nm)
A	0	0	–	2.5
B	9.9	52.3	35.1	3.1
C	10.7	56.5	35.8	3.6
D	12.8	67.6	36.3	7.5

**Fig. 4:**
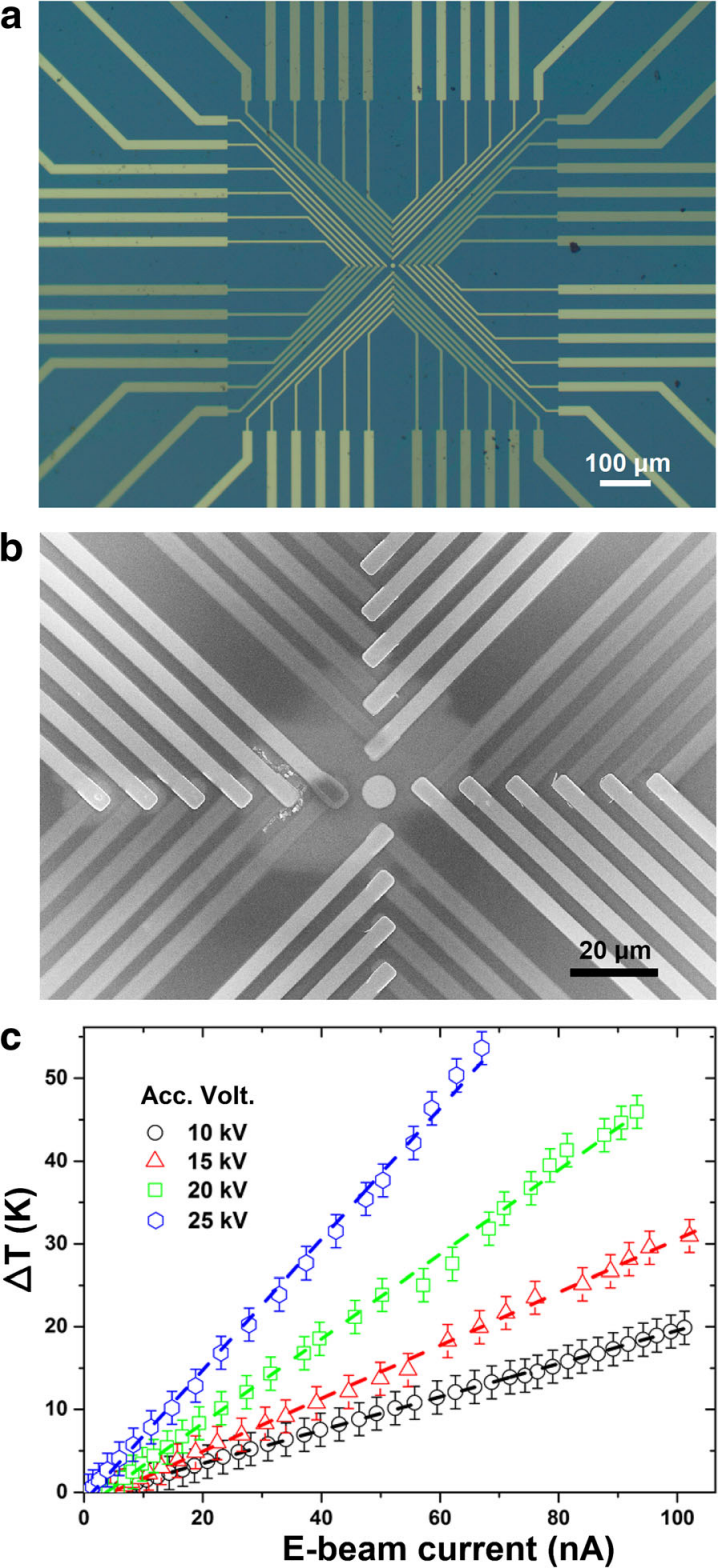
Images TFTC samples in Si and the testing results. **a** Optical image of a TFTC array on Si with junction size of 5.0 × 5.0 μm^2^. **b** SEM image of the device center, showing 24 sensor junctions. **c** Measurement results of local temperature increment with the TFTCs under e-beam irradiation with different accelerating voltages and beam currents

When the intensity of an incident e-beam is further reduced, or the irradiation duration is reduced, an e-beam causes measurable local heating effect at the surface under the irradiation. Figure 4 presents a set of typical measurement results. Figure 4a is an optical image for one of the four kinds of TFTC array samples developed in this work. Made on a 400-μm-thick SiO_2_/Si(100) substrate, this device consists of 24 identical Pt-Cr TFTCs. The 24 junctions, each with an area of 5.0 × 5.0 μm^2^, range in 4 rows, making a “cross” pattern in the center of the image. Figure 4b is a SEM image showing the center of the device, where the brighter beams are the Pt stripes, and the darker ones are Cr stripes.

Figure 4c plots some measured data. They were taken from different TFTC sensors under e-beam irradiation at different accelerating voltages of 10, 15, 20, and 25 kV, respectively, with increasing beam currents. The beam spot diameter was fixed at 1 μm and the measurement time was fixed at 30 s. Calibration experiments revealed that the average sensitivity of Pt-Cr TFTCs were 15.00 ± 0.29 μV/K, with a relative standard deviation of 1.9%. It shows that the local temperature increment linearly increases with the incident e-beam current. This indicates a linear surface heating effect, that the heating power converted to the local subject surface (here the TFTC junction) was proportional to the electron flux in the incident e-beam. Within the measurement error, the heating power was also proportional to the acceleration voltage. However, as we will discuss in the following, the measured data were much lower than the exact local temperature increment at the center of the e-beam.

## Discussions

### Centralized Distribution of Local Temperatures Under a Nanoscale e-Beam

**Fig. 5:**
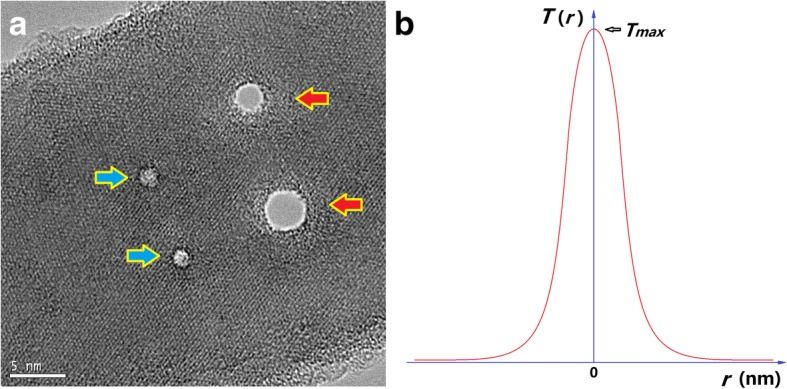
Distribution of local temperature under irradiation of a nanoscale e-beam. **a** A single crystalline Si NW with four nano-holes drilled with a HIEB in TEM. Two shallow holes are highlighted with blue arrows, and two through holes are highlighted with red arrows. **b** Estimated *T*(*r*) function for the local temperature versus distance to the central point

We noticed that when a NW was irradiated with a nanoscale e-beam, the central region had a much higher temperature than the rest region. Figure 5a shows a single crystalline Si NW with four nano-holes drilled in TEM with a HIEB. The two left holes, highlighted with blue arrows, have a diameter of 1.2–1.5 nm, and they are shallow holes, which do not penetrate through the NW. The two right holes, highlighted with red arrows, are through holes with diameter of 2.5 nm and 4.0 nm, respectively. It is clearly shown that a nano-ring region around the hole show amorphous structure, while the remaining part of the NW retains its original crystalline structure. For instance, the central region between two neighboring holes, which is only 2–4 nm away from the hole edge, shows clear crystalline periods along two directions.

This phenomenon implies that under a nano-e-beam, the center of the subject under irradiation has a highest temperature, and away from this center, the local temperature decreases sharply. Figure 5b schematically illustrates the assumption: The *T*(*r*) function is similar to a delta-function, where *T* is the local temperature and *r* is the distance from the central point of the e-beam. We may also assume that when the e-beam increases its diameter, there is a plateau in the middle of the *T*(*r*) curve, where the local temperature saturates, and when the beam diameter further increases, the plateau increases its saturation area.

**Fig. 6:**
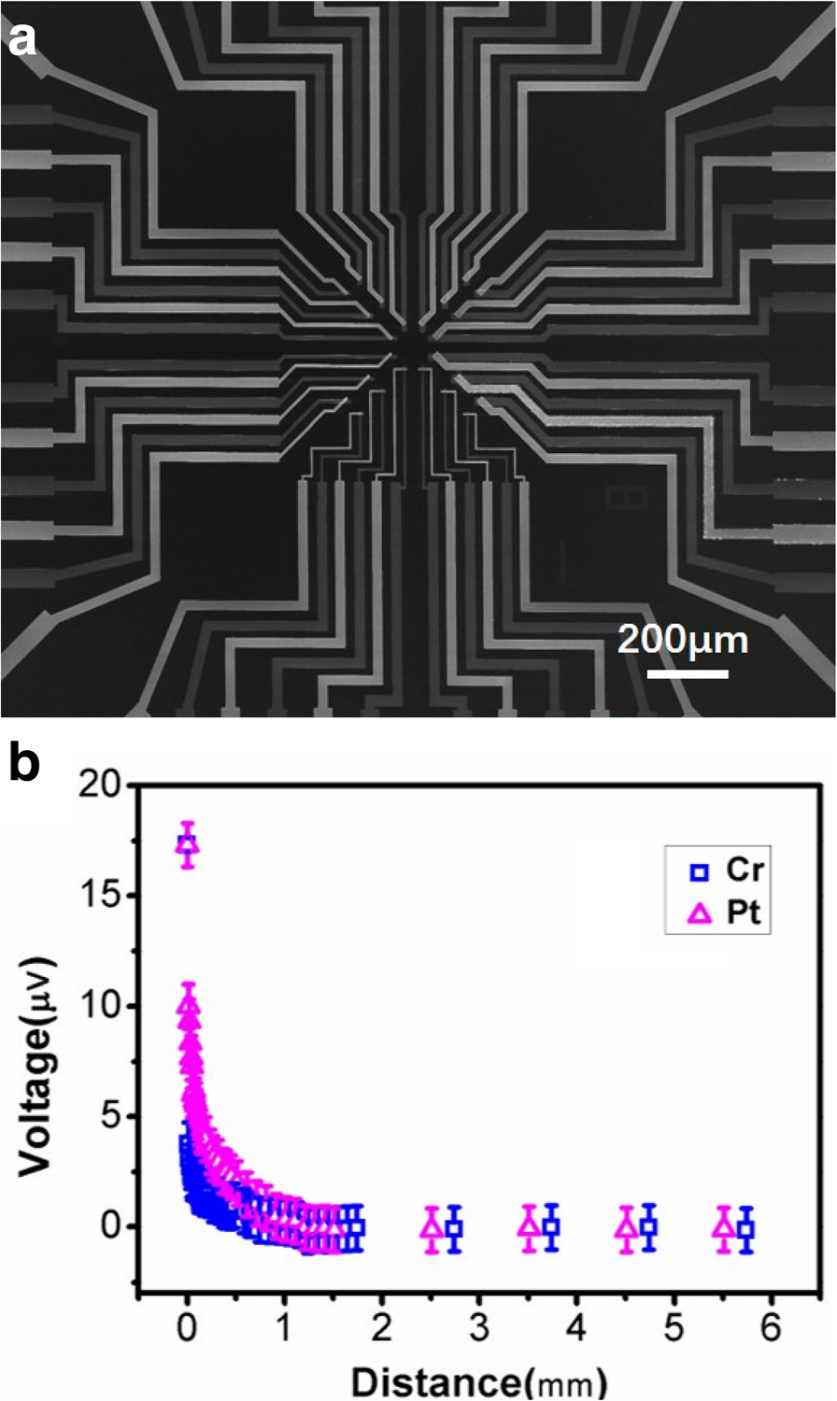
A TFTC array and its measurement results. **a** SEM image of a Pt-Cr TFTC array sample on the front surface of a Si_3_N_4_/Si(100)/Si_3_N_4_ wafer. The TFTC array on the central of the device consisted of 24 TFTCs, which had junction size ranged from 2.0 × 2.5 to 8.0 × 8.5 μm^2^. **b** Measured outputs from one TFTC sensor when a focused e-beam of diameter 1 micro was irradiating on a spot of the two metallic thin film stripes of the TFTC, namely Pt and Cr, at certain distance to the Pt-Cr junction region

In addition to the observations in TEM as typically shown in Fig. 4a, the above assumption of local temperature distribution was tested qualitatively with our TFTC arrays under a weak e-beam in SEM. Figure 6a is a front-side SEM image for a Pt-Cr TFTC arrays made on a Si wafer. In Fig. 6b, we present results of a unique measurement. The data were obtained under the condition that the e-beam was focused to have a beam size of 1 μm, while the focal point of the e-beam was moved in situ on the Pt or Cr beams of each Pt-Cr TFTC sensor. The distance from the focal point to the TFTC junction region was carefully measured. Under this experimental setup, we assumed that the local temperature of the focal point was almost the same, while the measured data differed remarkably as the distance changed. The results showed that after a distance of 1.5 mm from the junction region, the thermal effect caused by the focal e-beam was almost negligible.

### Influence of Sensor Size and on the Measurement Results

**Fig. 7:**
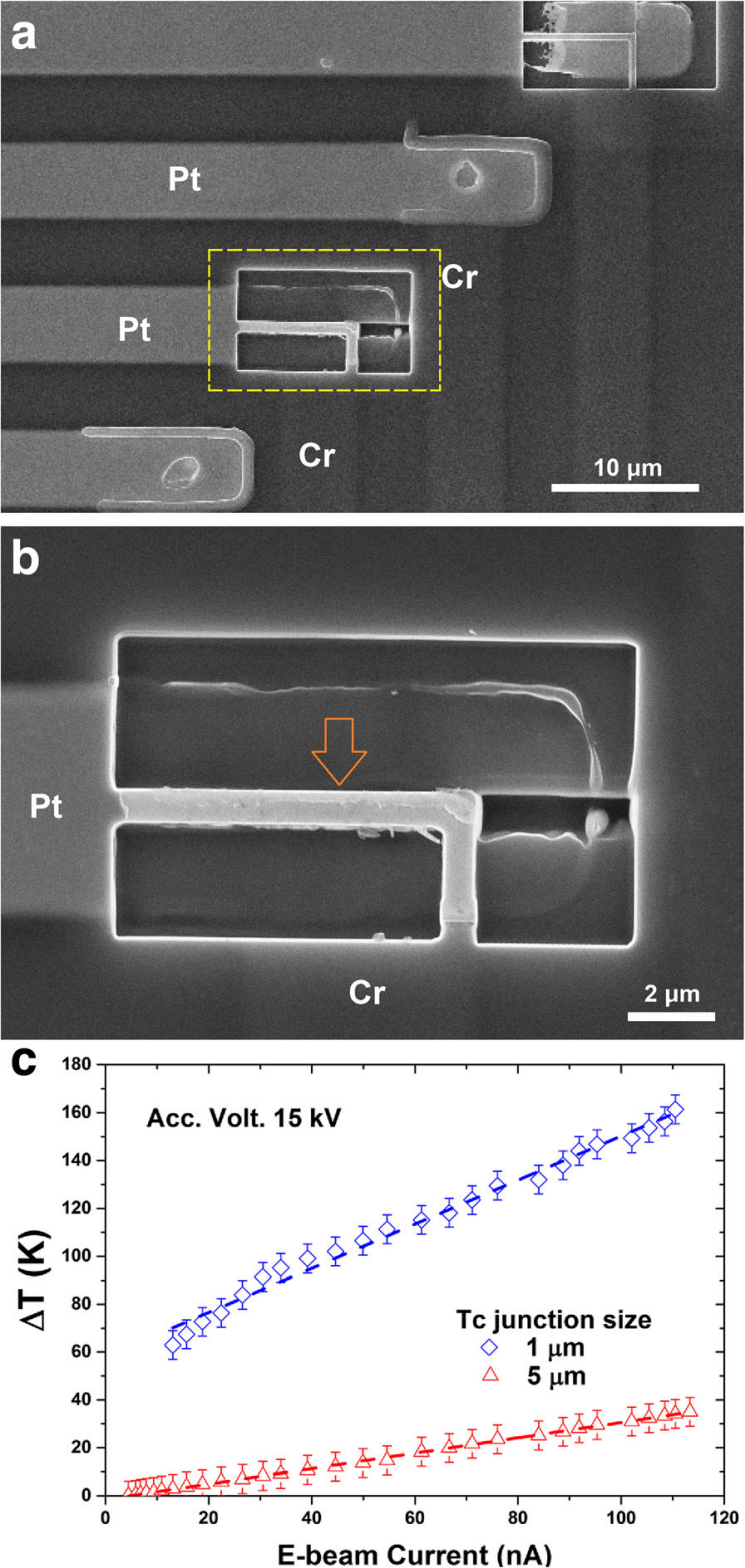
SEM images of a TFTC array and its measurement results. **a** SEM image of a Pt-Cr TFTC array on thick Si wafer with identical original junction size of 5.0 × 5.0 μm^2^. One junction (highlighted with a dashed yellow frame) was cut with FIB to a junction area of 1.0 × 1.0 μm^2^. **b** SEM image of the FIB fabricated junction area in (**a**). **c** Measured outputs from an original TFTC and the small junction TFTC under the same e-beam irradiation

We found that the junction size of our TFTCs had a crucial influence on the measurement results. On a Si with 24 Pt-Cr TFTCs, the original junctions had a size of 5.0 × 5.0 μm^2^. We used focused ion beam (FIB) technique to make some of the junctions into a smaller junction size of 1.0 × 1.0 μm^2^, as shown in Fig. 7a, b. Under the same irradiation of e-beams, the outputs taken from the TFTC with a small junction size of 1.0 × 1.0 μm^2^ were much higher than those with junction size of 5.0 × 5.0 μm^2^, as shown in Fig. 7c. For example, irradiated with an e-bema of accelerating voltage 15 kV and beam current 113.3 nA, the TFTC with junction size of 5.0 × 5.0 μm^2^ measured an local temperature increment of 35.0 K. Under the same condition, an increment of 161.4 K was measured by the TFTC with junction size of 1.0 × 1.0 μm^2^, enhanced five times. Again, it confirmed that the peak surface temperature under a nano-microscale e-beam was localized in a very small area.

### Influence of Freestanding Si_3_N_4_ Window on the Measurement Results

The substrate for our TFTC sensor plays an important role on the maximum local surface temperature of a subject under irradiation of nano-microscaled e-beams. When the substrate is thick, heat dissipated through the substrate may be much more than the local heat accumulates on the subject surface. As a result, the measured local temperature could be much lower than the possible *T*_max_ induced by the incident e-beam.

Generally, an incident nano-micro-scaled e-beam generates an amount of local heat, *Q*, at the surface of the subject under irradiation, which can be described by *Q* = *P*·Δ*t*·*γ*, where *P*·is the incident kinetic power, Δ*t* is the time duration, and *γ* is the converting ratio. Ignoring the relativity effect, roughly *P*·Δ*t*·*γ* = *I*·*V*·Δ*t*·*γ*, where *I* is the beam current, and *V* is the accelerating voltage. Some heat is expected to dissipate though the substrate, TFTC leads, and radiation. The remaining part causes increment of the local surface temperature that is measurable by the TFTC sensors. That is, *Q* = *Q*_substrate_ + *Q*_lead_ + *Q*_radiation_ + *Q*_sensor_, and *Q*_sensor_ = *C*·Δ*T* + *λ*. Here, *Q*_substrate_, *Q*_lead_, and *Q*_radiation_ represent thermally dissipated heat through the substrate, sensor leads, and radiation effect, respectively. *Q*_sensor_ corresponds to the remained heat measured by the sensor, *C* is the thermal capacity of the sensor junction, Δ*T* is the increment of local temperature as compared to the cold ends of TFTCs, and *λ* is the latent heat of phase transition. Our previous studies have shown that, under the irradiation of the same e-beams, the measured output from TFTC sensors made on freestanding Si_3_N_4_ window was 10–30 times larger than that taken from the same sensors on thick Si wafers [40]. This factor of enhancement indicated that *Q*_substrate_ was much larger than *Q*_sensor_.

We noted that the converting ratio *γ*, or referred as thermal efficiency value in some publications, depended very much on the average kinetic energy. The converting ratio *γ* of a welding electron beam with an accelerating voltage of 70 kV had been revealed in the range of 0.33–0.48, and it was found that this ration had little correlation with the weld geometry [48]. Experiments had manifested that under low energy high current pulsed electron beam (LEHCPEB) irradiation, a homogeneous layer had been formed on the surfaces of steels, which could improve the anti-corrosion properties of steels dramatically [49]. However, in our TEM experiments, the *γ* values were extremely lower than one unit by several orders of magnitude. The underlying physics need further investigation.

**Fig. 8:**
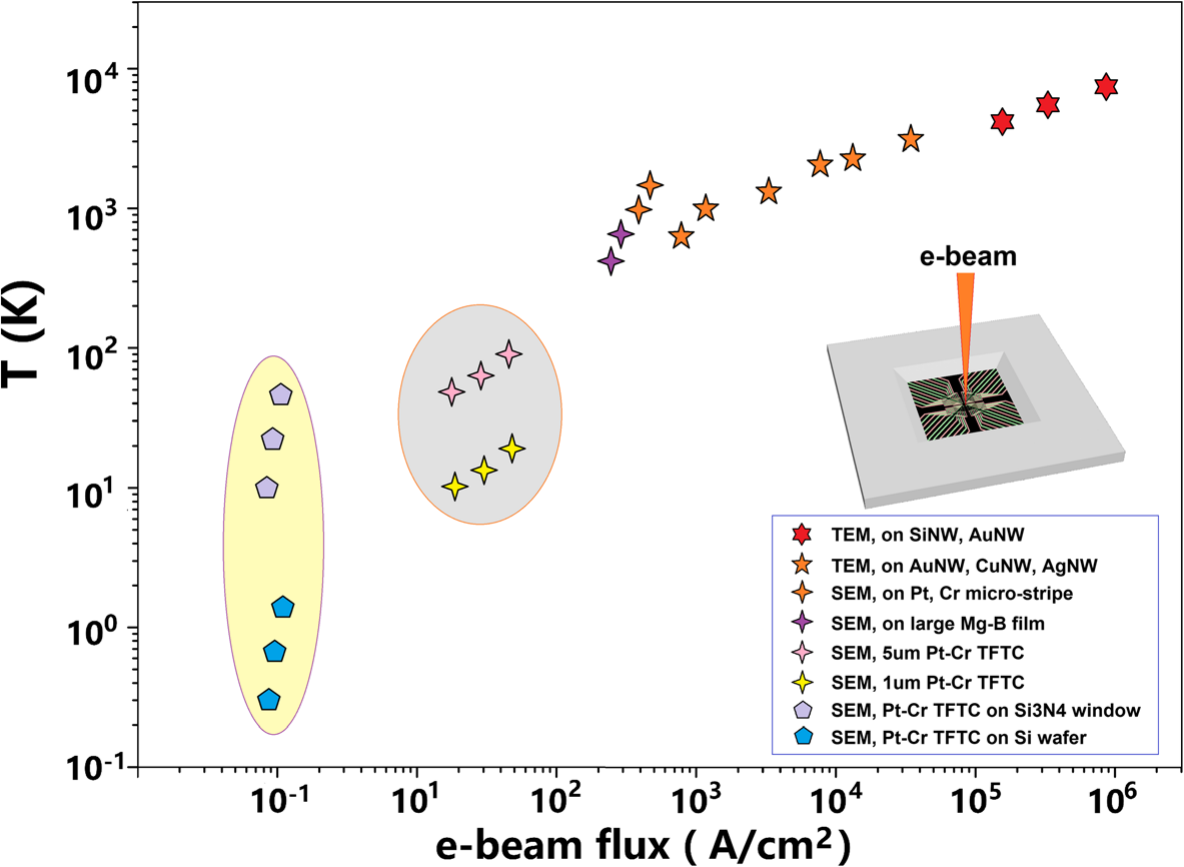
An illustration for the overall picture of the nominal local temperatures under irradiation of nano-/micro-scale e-beams. The gray oval indicates the comparison between small and large TFTCs on thick Si wafers. The yellow oval indicates the comparison between TFTCs on thick Si wafers and on freestanding Si_3_N_4_ thin film windows. For *T* > 1500 K, the data points are estimated values from morphology or phase change

We summarize the results and discussions of this work in Fig. 8. Our experimental results taken from irradiation of high-intensity e-beams in either a TEM or a SEM were consistent with each other. A local surface *T*_max_ higher than 1800–2000 K was achievable in both TEM and SEM, as revealed by local meting phenomena observed in Au NWs, Cu NWs, Pt, and Cr thin film stripes. In TEM, since the nanomaterials investigated in this work were naturally freestanding, *T*_max_ higher than 3000 K was evaluated from the observed nano-drilling experiments on Si NWs and Au NWs. Local vaporization effects were induced in seconds by 1 nm diameter e-beams.

We showed that TFTC on a freestanding Si_3_N_4_ thin film window resulted in an enhanced sensitivity by a factor of 10–30 times, and we showed that a 1-μm-wide TFTC sensor had a sensitivity higher than that of a 5-μm-wide TFTC by a factor of five times. This is because thick substrate and wide leads both dissipated a large amount of local heat. For precise measurement of local surface temperature at the nano- and micro-scales, ideally one should fabricate TFTC sensors as small as possible, and make them on thermal isolation layers, such as freestanding Si_3_N_4_ thin film windows or Parylene layers.

## Conclusion

In summary, we investigated several approaches for the measurement and estimation of local surface temperature under irradiation of nano-micro-scale e-beams. E-beams of 10^5-6^ A/cm^2^ could induce local vaporization of Si and Au in seconds, showing a temperature higher than 3000 K. E-beams with intensity of 10^3-4^ A/cm^2^ could introduce local melting in Cr, Pt, and Pd thin film stripes; Au and Cu nanowires; and phase transition in Mg-B thin films, with a local temperature of 1000–2000 K. We demonstrated that TFTC arrays made on freestanding Si_3_N_4_ windows worked well in detecting peaked temperature up to 1500 K or higher. By combining analysis techniques of surface morphology, electrical measurement, and TFTC sensors, we could estimate the local temperature in a wide range. We also discussed the distribution of surface temperatures under e-beams, thermal dissipation of thick substrate, and a small converting ratio from the high kinetic energy of e-beam to the surface heat.

The results are helpful for applications of e-beams, and may offer valuable clues for developing novel sensing techniques and evaluation methods for high temperatures in the range of 1500–3000 K.

## Abbreviations

AFM: Atomic force microscope
EELS: Electron energy loss spectroscopy
FIB: Focused ion beam
HIEB: High-intensity e-beam
LPCVD: Low-pressure chemical vapor deposition
NW: Nanowire
SEM: Scanning electron microscope
STEM: Scanning transmission electron microscope
TEM: Transmission electron microscope
TFTC: Thin film thermocouple
UHV: Ultra-high vacuum
